# Genetic Network and Breeding Patterns of a Sicklefin Lemon Shark (*Negaprion acutidens*) Population in the Society Islands, French Polynesia

**DOI:** 10.1371/journal.pone.0073899

**Published:** 2013-08-13

**Authors:** Johann Mourier, Nicolas Buray, Jennifer K. Schultz, Eric Clua, Serge Planes

**Affiliations:** 1 LabEx «CORAIL» - USR 3278 CNRS-EPHE, Centre de Recherche Insulaire et Observatoire de l’Environnement (CRIOBE), Papetoai, Moorea, French Polynesia; 2 National Marine Fisheries Service, Silver Spring, Maryland, United States of America; 3 Délégation Régionale à la Recherche et Technologie, Haut-commissariat de la République française, Papeete, Tahiti, Polynésie Française; 4 Ministère de l’Agriculture et de la Pêche, Paris, France; CNRS, Université de Bourgogne, France

## Abstract

Human pressures have put many top predator populations at risk of extinction. Recent years have seen alarming declines in sharks worldwide, while their resilience remains poorly understood. Studying the ecology of small populations of marine predators is a priority to better understand their ability to withstand anthropogenic and environmental stressors. In the present study, we monitored a naturally small island population of 40 adult sicklefin lemon sharks in Moorea, French Polynesia over 5 years. We reconstructed the genetic relationships among individuals and determined the population’s mating system. The genetic network illustrates that all individuals, except one, are interconnected at least through one first order genetic relationship. While this species developed a clear inbreeding avoidance strategy involving dispersal and migration, the small population size, low number of breeders, and the fragmented environment characterizing these tropical islands, limits its complete effectiveness.

## Introduction

Human population growth has fragmented the range of many species [1], leading to significant declines in abundance, loss of genetic diversity and an elevated risk of extinction [2,3]. Such patterns are mainly reported from terrestrial systems [4], but marine systems are now showing similar trends [5]. Isolation and reduction in population size erode evolutionary potential and raise the risk of extinction through inbreeding depression, leading to the loss of genetic variation or the accumulation of deleterious alleles [3,6]. To this end, many species have developed inbreeding avoidance strategies [7], although some animal populations naturally show low genetic variability [8,9].

Globally, sharks are threatened by overfishing [10–12]. While these threats are evident for pelagic species [13] as by-catch of commercial fisheries, populations of reef-associated species are also declining [11,14]. Sharks appear to be particularly vulnerable to over-exploitation because of their K-selected life-history strategy (i.e., slow growth, late sexual maturity, long life spans and low fecundity). As a result, overfished populations may require several decades to recover [10,11,15]. Chronic overfishing of sharks has diminished population sizes, fragmented large populations into small, locally-isolated ones [14], and led to trophic cascades [16]. Despite these rapid declines, little is known about their ability to persist in smaller, more fragmented populations and to recover from human-induced bottlenecks [17]. In addition, there is a lack of information about the fine-scale population genetic structure and breeding patterns of most reef shark species (but see 18–21). As top predators, sharks frequently exhibit small population sizes and are therefore a good model to investigate the interactions between demographic parameters, genetic population structure, mating system and levels of inbreeding in natural small marine populations, especially in isolated insular systems.

For 5 years, we monitored a small sicklefin lemon shark ( *Negaprion* *acutidens* ) population around Moorea, French Polynesia [22]. This species is a large (≤ 340 cm total length) coastal shark that occurs in the Indo-Pacific region [23]. The species is now listed as globally vulnerable on the IUCN Redlist. It is a viviparous shark giving birth every second year on average, between August and October, to 1-13 well-developed pups after a 10-11 month gestation period [23]. The small population of Moorea [22] has not experienced a high level of exploitation, as sharks are not of commercial interest or traditionally fished locally, and sharks in French Polynesia have been formally protected by law since 2006. Low densities may therefore be a result of natural ecosystem equilibrium in an isolated, fragmented, insular system and/or due to some recent natural bottleneck [24]. Meanwhile this species supports part of the diving industry in French Polynesia with special feeding dives organised [22,25].

The aim of this study was to describe genetic relatedness and assign parentage based on microsatellite DNA markers in order to investigate the genetic makeup and mating output within a small isolated shark population. Previous parentage analyses of sharks were mostly based on the reconstruction of parental genotypes from sampled offspring and, when possible, included few opportunistic sampled adults in the analysis [18–20]. The present approach differs since it combines a 5-years monitoring of the population in Moorea together with a genetic analysis of relationships among individuals within and between generations.

## Methods

### Ethics Statement

All necessary permits were obtained for the described field study from the French Polynesia Ministry of Research to the authors. No specific permission was required for underwater surveys as they were conducted at a commercial diving site and only involved photo-identification surveys (see below). Every shark species is protected under French Polynesian laws, however, DNA samples were taken by non-invasive methods approved and conducted under the French Polynesia Ministry of Research permitting authority (Permit # 653/MRM/SPE/DEV), and no lethal sampling was conducted. Juvenile sharks were captured with gillnets and released in the water alive under good conditions and adults were remotely sampled with a modified speargun with a biopsy tip.

### Study background and tissue collection

Our study was conducted in French Polynesia, a fragmented system of small islands and atolls separated by deep ocean (depth ≥ 2 000 m), which is expected to increase the effective isolation of reef-associated populations [26]. We monitored a population of sicklefin lemon sharks ( *Negaprion* *acutidens* ) visiting a recreational diving site during a 5-year period in Moorea (17° S, 149° W; Figure S1). Diving surveys were implemented on a daily basis representing 1058 days between January 2005 and September 2009 [22]. Based on photo-identification [27], we consistently identified 40 mature sharks (18 males and 22 females ranging from 2.4 to 3.1 meters total length), with an average of 26.75 ± 3.33 individuals sighted per year (24 in 2005, 28 in 2006, 31 in 2007, 29 in 2008 and 24 in 2009). When possible, a fin clip was removed from the dorsal fin of each new shark using a modified spear gun. As a result, 29 out of 40 individuals (72.5%) were sampled. All 11 non-sampled sharks were observed only once or very few times and disappeared before sampling. Additionally, 4 resident females were sampled in Bora Bora (230 km from Moorea) where only 12 females (but no males) have been observed. From this survey, all sharks were classified into behavioural groups based on the affinity between sharks and their fidelity to the site [22] and were assigned to three categories:

• *Resident sharks of Moorea*: composed of 7 males (M03, M04, M05, M07, M10, M18 and M31) and 7 females (F08, F11, F15, F20, F23, F25 and F29);
• *Non-resident sharks visiting Moorea occasionally*: composed of 6 males (M09, M12, M19, M34, M36 and M38) and 9 females (F01, F02, F06, F13, F17, F21, F26, F27 and F30);
• *Bora-Bora resident sharks*: composed of 4 females only sighted in Bora Bora (B1, B2, B3 and B4).

In addition to adults on Moorea and Bora-Bora sites we also captured 52 newborn and immature sharks from different cohorts between 2006 and 2009 in Moorea and three neighbouring islands: Tetiaora (40 km), Tahaa (200 km) and Rangiroa (330 km) (Figure S1). Annual sampling of juveniles took place soon after pupping by adult females (between December and February). Most newborn sharks (or age-0) could be identified based on the presence of an open (or recently closed) umbilical scar that closes few months after birth. The age of sharks without umbilical scars was determined based on body length, whose distribution is generally non-overlapping between age-0, age-1 and age-2 (85<age-1<100 cm TL, 100<age-1<115 cm TL and 115<age-1<130 cm TL, respectively, as determined based on ongoing growth calculations from capture-recapture data). This was also confirmed with recapture of individuals in two consecutive years. Sharks that were larger than 130 cm TL were assigned to an unknown year of birth (‘other’). Tissues were stored in 95% ethanol.

### Underwater observations of reproductive status

During our underwater photo-identification surveys, we reported the presence of dermal bite wounds and scars on females’ body as a sign of mating activity [21,28]. Courtship behaviour with males engaged in close following of females was also reported with identification of male and female ID when possible [29]. Finally, the period of parturition was estimated based on the reappearance of newly slender female after the pregnant female left the site for several days or weeks [21,28].

### DNA isolation and microsatellite genotyping

Genomic DNA was isolated from each fin-clip using a DNA Purification Kit (Puregene). We isolated 2 species-specific polymorphic microsatellite loci from *Negaprion* *acutidens* and selected 14 polymorphic microsatellite loci developed for other shark species (Table S1). All specimens (n = 85) were genotyped at all 16 polymorphic microsatellite loci. PCRs were conducted with forward primers labelled with Beckman Coulter dyes D2, D3 or D4 (Table S1). Amplified fragments were separated on a Beckman Coulter CEQ 8000 Genetic Analysis System, with a 400-bp internal size standard. Genotypes and allele sizes were scored using Beckman-Coulter CEQ TM 8000 Genetic Analysis System-associated software. Allelic frequency and expected heterozygosity under Hardy–Weinberg equilibrium were calculated for each locus in GENALEX 6 [30]. The presence of null alleles was investigated using MICROCHECKER [31]. Tests for Hardy-Weinberg and linkage disequilibrium were conducted in GENEPOP 3.4 [32] and significant levels were adjusted with sequential Bonferroni corrections for multiple tests with *P* < 0.05. Of the 16 microsatellite loci, three did not satisfy Hardy-Weinberg and linkage disequilibrium assumptions. To test our photo-identification technique [27], GENALEX 6 [30] was used to detect potential identical genotypes belonging to resampled individuals. No identical genotype was found confirming the accuracy of our photo-identification technique.

### Relatedness and genetic network construction

Maximum likelihood estimates of pairwise relatedness coefficients and genealogical relationships were calculated with the software ML-RELATE [33] computing 5000 iterations. The program calculates the maximum likelihood relationship between individual pairs. It determines which of parent-offspring (PO), full-sibling (FS), half sibling (HS) and unrelated (U) categories yields the greatest likelihood.

Instead of trying to reconstruct an exact pedigree that is challenging from genetic data alone in wild populations, we built a genetic network using relatedness information [34]. Network analysis is now a common tool used to characterize animal social associations [35] including sharks [36]. However, it has rarely been used to illustrate the genetic relationships between individuals in a population despite its advantage to incorporate a large amount of data into a simple visual graph [34]. The genetic network was built from the matrix of genetic relatedness together with individual characteristics (size, sex and group membership) using the programs SOCPROG [37] and NETDRAW 2.123 [38]. Only the R values of first-order genetic relationships (PO, FS and HS) inferred from ML-RELATE were retained in this network for an easier visualization.

### Parentage analysis

The program CERVUS [39] was first used to find highly probable mother–offspring and father–offspring pairs. Assignment to these potential parents was done under a strict confidence level of 95%. These mother–offspring and father–offspring pairs were then identified in the input file of COLONY 2.0.3.0 [40] as known maternity and paternity. COLONY 2.0.3.0 [40] implements a full-likelihood approach to parentage analysis and was shown to outperform other programs in cases of less than 20 microsatellite markers [41]. We considered both parent-offspring relationships and sibship amongst offspring. Adults were separated by sex, and we assumed a polygamous mating system for both sexes, therefore allowing the assignment of half-siblings. We carried out a long-run with medium likelihood precision and a genotyping error rate of 1%. The prior probability that the true parent was present in the sample was set to 0.5 for fathers and 0.25 for mothers based on the proportion of sampled males and females that were assigned to offspring by CERVUS. We conducted other runs varying the input parameters such as the mating system for each sex. For each analysis, 3 replicate runs were conducted on the same data set. Each of the replicate runs used different random number seeds to initiate the simulated annealing processes. Parental genotype reconstruction was also performed with COLONY allowing to infer the number of breeders in the population. Therefore, total number of breeders was assessed either by reconstructing males and females’ genotypes from young of the year half-sib groups or by directly assigning parentage to offspring from sampled adults.

### Inbreeding estimations

To test for inbreeding, we used a Monte Carlo simulation implemented in STORM 1.0 [42]. This analysis generates offspring internal relatedness (IR) values expected from the gene pool if mating is random with respect to parental relatedness. We calculated the average observed IR for all offspring (n = 52) and adults (n = 33) sampled. We generated simulated IR measures by sampling random males (n = 13) and females (n = 20) with replacement. Each random mating pair produces a simulated offspring whose internal relatedness can be measured. The observed mean IR was then compared with the distribution of average simulated IR produced from 1000 iterations. Potential bias of allelic diversity into IR estimates was tested by resampling and recalculating IR values on loci with 5 or more alleles.

## Results

### Microsatellite summary statistics

Across all individuals of *Negaprion* *acutidens* collected for this work, polymorphism varied from 2 to 15 alleles per locus. Observed heterozygosity ranged from 0.365 to 0.871 per locus and expected heterozygosity from 0.395 to 0.871 (Table S1). Null alleles were detected at loci LS15 and Cli107, as suggested by the general excess of homozygotes using MICRO-CHECKER. Overall, significant heterozygote deficiencies were found in three loci (LS15, Cli107 and Cpl169; with all P < 0.05 following Bonferroni standard correction). We therefore decided to remove both LS15 and Cli107 from subsequent analyses due to evidence of null alleles. Once analyzing together the remaining 14 loci, no significant heterozygote deficiencies were observed in the global population (P>0.05).

### Adult population genetic structure

**Figure 1:**
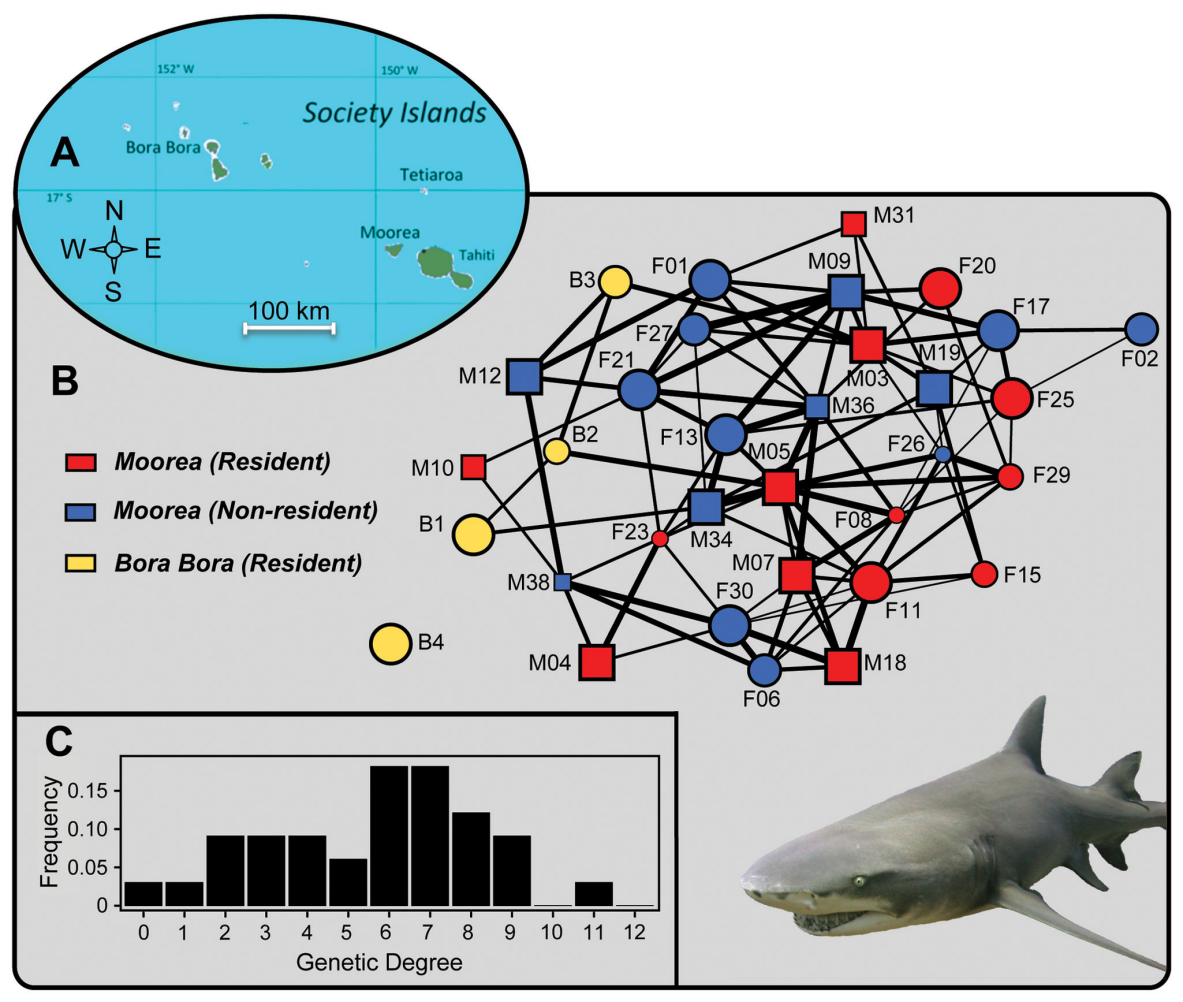
Genetic network of the sicklefin lemon shark population from Moorea and Bora Bora. (A) Map of the study location. (B) The genetic network of adult lemon sharks. Each individual is indicated by a node labelled by shark ID. Circles and squares indicate females and males respectively and symbol size is indicative of the body length of the shark. Node colour corresponds to the three defined residency groups. Dyads sharing a first-order genetic relationship are connected by a line, with line thickness indicating the strength of the genetic relationship (proportional to R values). A ‘spring embedding’ algorithm with node repulsion for laying out the nodes’ positions [38] was used to cluster densely connected nodes together with less connected nodes placed around the edge. (C) Genetic degree (number of first-order genetic relationships an individual has) distribution within the population.

As expected in a wild population, the mean coefficient of relatedness was low among adults, both within the overall population of Moorea (mean R ± SD = 0.086 ± 0.137) and when we included individuals from Bora Bora (mean R ± SD = 0.076 ± 0.128; Table S2). However, the genetic network illustrates that all individuals are interconnected at least by one first order genetic relationship except for female B4 from Bora Bora that is isolated from the network (Figure 1). First order genetic relationships (PO, FS and HS) accounted for 21.6% of all pairwise relationships in Moorea and 17.6% when individuals from Bora Bora are included (Figure 1). The number of connections a focal individual has in the network (genetic degree) ranged from 0 to 11 among the 32 other members of the network (mean ± SD = 5.6 ± 2.6; Figure 1C), indicating that individuals have a high number of close relatives in the population. Residency groups as well as sex categories were also all genetically linked (Figure 1B). Within-group average genetic relatedness was significantly different from between group (*P* < 0.001) even when excluding resident sharks of Bora Bora (*P* < 0.05; Table S2). There was no difference between sex (*P* > 0.05; Table S2).

### Visual information on reproductive status

**Figure 2:**
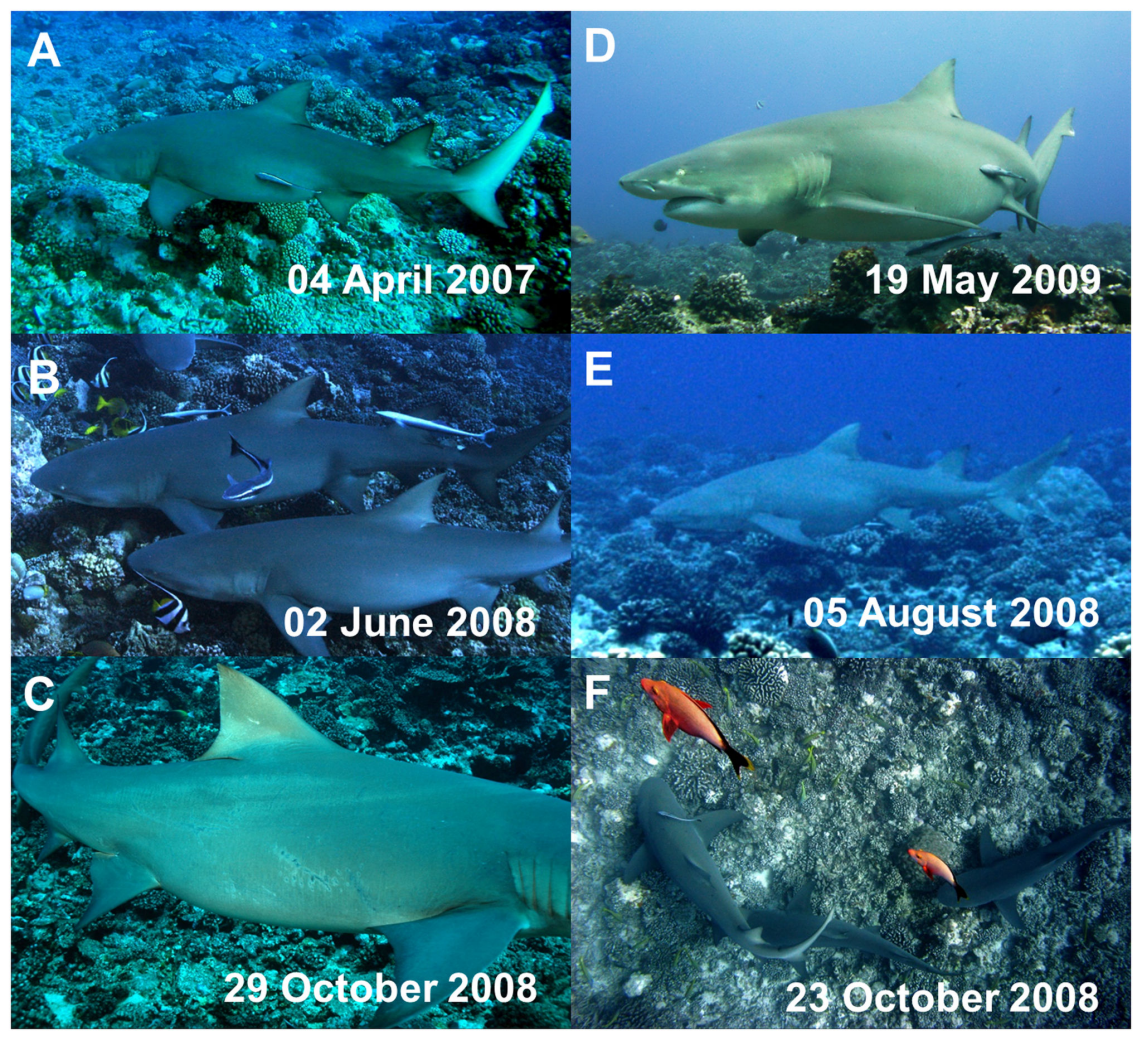
Inference of reproductive cycle from underwater surveys. (A–D) A two-year reproductive cycle as displayed by female F11 which was pregnant in 2007 (A), then entered in a resting period (B) and mated in 2008 as shown by dermal bite wounds on its flanks (C), and was pregnant again in 2009 (D). (E–F) Female F01 is pregnant in 2008 (E) and is followed by males M10 and M31 in a courtship behavior just after parturition in 2008 (F).

Pre-copulatory and courtship behavior, characterized by a male showing a close nose to tail behavior while following a female (Figure 2F; Table S3; Video S1), started in August and lasted until early November (first observation in August 20^th^ and last observation in November 1^st^), with a stable calendar each year. Several males can be involved in courtship behavior at the same time following the same female (Figure 2F; Table S3). Females with specific mating scars were then observed from the end of September throughout November (Figure 2C; Table S4). Pregnancy in females became visually apparent in February and progressed until being fully apparent in May (Figure 2). Most females followed a 2 years reproductive cycle (Figure 2A–D) with the exception of females F01 (Figure 2E) and F21 that became pregnant on 2 consecutive years (Table S4). Considering the reappearance at the site of newly slender females together with first newborn sharks found in their nursery in October, we determined that parturition occurs between July and November (Figure 2E-F; Table S4). Therefore, gestation is estimated to last 10 to 11 months.

### Parentage analysis

**Figure 3:**
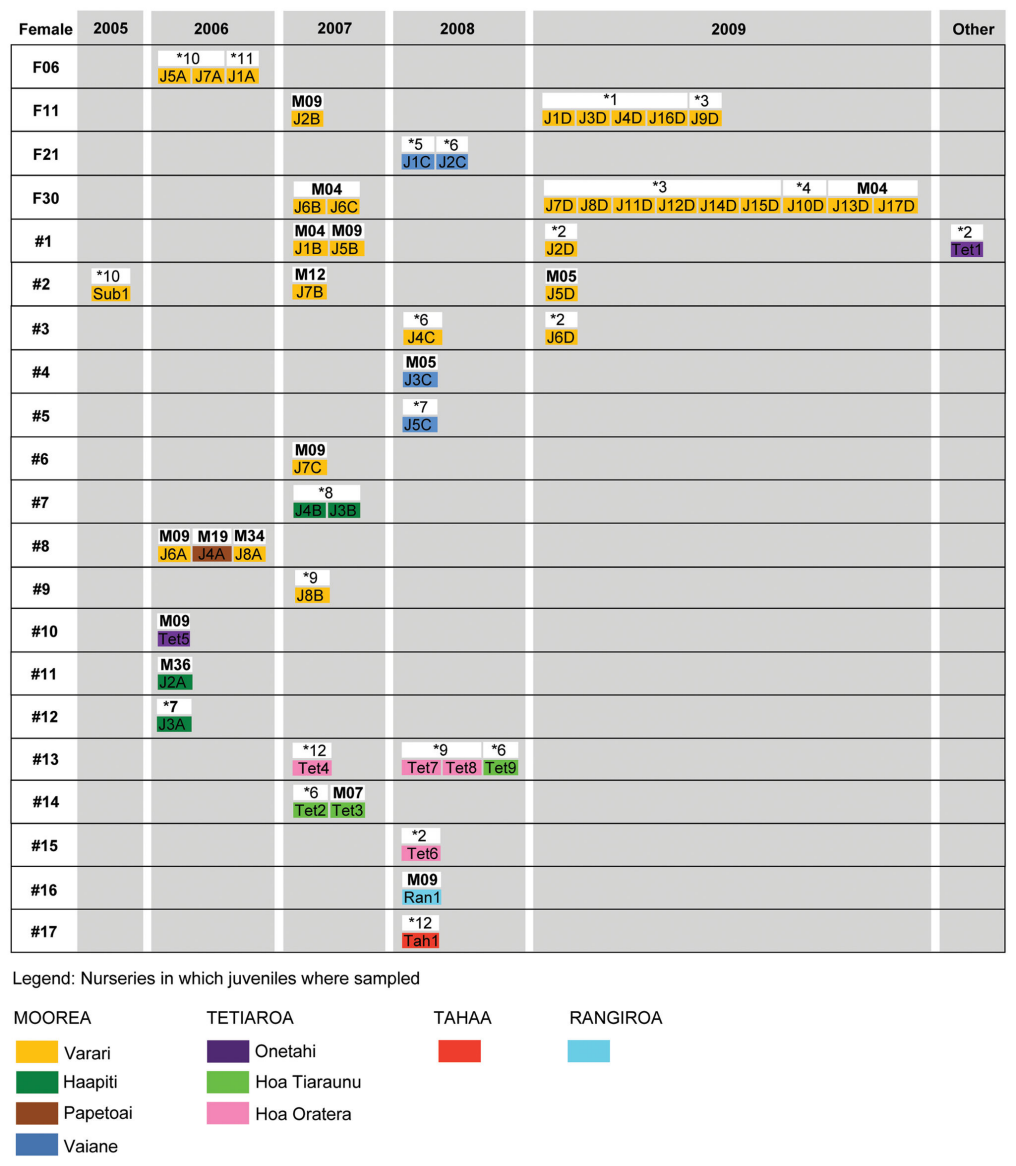
Female reproduction inferred from parentage assignment. Litters are shown by years for each female with the assigned father(s) and juvenile(s). Sampled individual adult sharks are indicated in bold. Mothers and fathers inferred through genotype reconstruction by the program COLONY are identified by #ID and *ID, respectively. Colour of juveniles refers to their sampling nursery site (Figure S1). Note that juvenile Tet5 was sampled in 2008 in Tetiaroa but was assigned to the birth year 2007 due to its size (110 cm) corresponding to an age-1 juvenile, while subadult Sub1 was sampled in 2008 at the size of 125 cm and subsequently assigned to year of birth 2006. Finally, Tet1 was assigned to an unknown year as we were not able to determine its year of birth.

Parentage analysis assigned 35 of the 52 genotyped juveniles to at least one parent or a parent pair among the 33 sampled adults (Figure 3). In all case, the three runs gave consistent results and mating system parameters did not consistently change parentage assignment results. Of the 35 juveniles, 17 (49%) were assigned only to a female, 13 (37%) only to a male and 5 (14%) to a parent pair. The two mating pairs of sampled sharks were F11-M09 (Resident/Non-resident) and F30-M09 (Non-resident/Non-resident). The 52 juveniles were assigned to 29 distinct litters across the years (Figure 3). From the adult sharks sampled (13 males and 20 females), only 4 females and 8 males were assigned to a juvenile. However, the genotype of 29 physically unsampled sharks (12 males and 17 females) was reconstructed by COLONY and assigned to a juvenile. No juvenile was assigned to a female from Bora Bora. Therefore, a total of 41 adult sharks including 20 males (8 sampled and 12 genetically reconstructed fathers) and 21 females (4 sampled and 17 genetically reconstructed mothers) contributed to the reproduction for our sampled juveniles (Table 1).

**Table 1:** Number of breeders determined by direct parentage assignment and genotype reconstruction conducted in program COLONY.

	Mothers	Fathers	Total
Physically sampled	4	8	12
Genetically reconstructed	17	12	29
Total (Ne)	21	20	41

### Female reproductive behaviour

Of the 21 females either sampled or genetically reconstructed (Table 1), 15 gave birth in Moorea and 4 in Tetiaroa (Figure 3). Six females returned to the same nursery on multiple years to give birth, of which 4 returned on a two-years cycle (F11, F30, #1 and #2; Figure 3) and 2 returned in consecutive years (#3 and #13; Figure 3). Two litters were made of juveniles sampled in different nurseries (Figure 3). Seven (78%) of the 9 litters cumulating more than one young were the result of polyandrous females with females mating with 2 to 3 males (Figure 3). Most parturition events confirmed the dermal bite wounds observed underwater (Figure 2) in the year prior to parturition (Table S4).

### Male reproductive behaviour

In our sample, 10 (50%) of the 20 fathers sired more than one litter (Figure 3). Five males mated with multiple (2,3) females in a single year (M09 in 2006; M04 and M09 in 2007; *6 in 2008; *2 and *3 in 2009). Nine males sired a litter in multiple years. Male M04 was assigned to offspring of two different litters of female F30 (Figure 3). Male M05 sired 2 different litters in 2 different years (2008 and 2009) although this shark has not been sighted in Moorea since early 2006.

### Genetic diversity and inbreeding

The mean IR value of offspring was not significantly higher than expected under random mating (Monte Carlo randomization (x1000): mean IR ± 95% CI = 0.037 ± 0.056 and mean simulated IR ± 95% CI = 0.009 ± 0.001, *P* = 0.142). IR values calculated from loci with over 5 alleles were not different from those calculated from all loci (mean IR ± 95% CI = 0.023 ± 0.047; P > 0.05) suggesting that our estimation of IR values were not inflated by the presence of low allelic diversity at some loci. Observed maximum IR value was 0.504, 13% (7/52) of all offspring had IR values higher than 0.25 (the value expected for offspring of half-sibling mating), and 36% (19/52) had o-IR larger than 0.125 (Figure S2).

There was a significant inverse relationship between body size (ca. age) and IR (y = -0.0005x+0.0780; *R* ^2^ = 0.0629; *P* = 0.011). This was confirmed by a significant difference in R values between maturity status (*F*_2,82_ = 3.248, *P* = 0.043); offspring IR values being higher than IR of mature sharks (*P* = 0.034; Figure 3A; Figure S2). Finally, offspring IR values did not vary significantly across years (*F*_3,46_ = 0.853, *P* = 0.472; Figure 3B).

## Discussion

This study provides a unique case of long term (5 years) monitoring and genetic survey used to construct the genetic network and to infer the mating output of an island adult shark population. This population is also unique since, unlike many places in the world, it has been preserved from any human exploitation, implying that low densities observed are normal for this population. Our results demonstrate that this small population displays patterns of connectivity throughout the archipelago and dispersal which are used as a strategy to avoid mating with closely related partners.

### Population genetic structure and dispersal

Although the mean relatedness was low among adults of the population, all sharks (but 1) were interconnected through close to moderate genetic relationships. First order genetic relationships accounted for 17.6% of all pairwise relationships, reaching 21.6% when excluding individuals from Bora Bora. The genetic network (Figure 1) illustrates that sharks from different residency groups and islands are related, that is likely a consequence of dispersal [37]. Our results also indicate that sharks are migrating to breed outside their resident population (Figure 2). While some individuals of its Atlantic sister species, *Negaprion* *brevirostris* , appear to remain at their natal island, others disperse before reaching maturity [43] to colonize other islands. A similar situation is found in *Negaprion* *acutidens* , as among four resident female sharks of Bora Bora, three (B1, B2 and B3) appeared genetically linked to sharks sighted in Moorea (resident M03 and M05; non-resident M12 and M34; Figure 1B). Also, direct migration evidence is available with some individuals from Moorea being sighted in Tahiti both within and outside the mating season (i.e. females F15, F20, F23, F25 and F30; Figure 1A). Moreover, during each breeding period (September–November), most resident males disappeared from days to weeks [22] while non-resident males, such as male M12, show up in in Moorea, presumably to mate (Figure 1B). While adult sharks appear to migrate throughout the archipelago, movement or dispersal in juvenile and immature sharks may be limited as only two age-0 juveniles were caught away from their littermates within the same island (female J4A about 8 km away in Moorea and Tet9 about 1.5 km away in Tetiaroa). However, female Tet1 (180 cm TL) caught in Tetiaroa is an offspring of female 1 that gave birth in Varari (Moorea) in 2007 and in 2009; therefore, this immature shark can either correspond to a dispersal event from its birth location or a change in nursery location of its mother (female 1) for parturition. The paucity of studies on *Negaprion* *acutidens* does not provide further information on the year of first dispersal (or emigration from nursery) of juveniles, but a recent study showed that among immature sharks (i.e., 141 < TL < 202 cm) the first sharks to leave the study area was about 150 cm TL [44]. Individual sharks have been shown to emigrate from their nursery during their third year in the sister species *Negaprion* *brevirostris* [43] and in their first year in *Carcharhinus* *melanopterus* [45]. However, some species delay their dispersal until reaching maturity [46]. Therefore, while sicklefin lemon sharks may start emigration from nurseries during their first year, dispersal rate in the population may increase throughout the shark’s growth [43]. Overall, sicklefin lemon sharks in the Society Islands appear to be structured as a mixed population of individuals moving throughout the archipelago (Figure 1; Figure 3).

### Reproductive behaviour

From parentage analysis, we were able to investigate the mating system and recruitment patterns of lemon sharks in the archipelago. Like other reef sharks depending on nurseries for recruitment [18,20,21], most females showed philopatry to particular nurseries (Figure 3) although this was not the case for all of them (Figure 3). *Negaprion* *acutidens* shares nursery locations with *Carcharhinus* *melanopterus* in Moorea and Tetiaroa [21,47]. As females mostly followed a biennial reproductive pattern, some did not exhibit a two-year cycle (Figure 2; Figure 3; Table S4). Therefore, females may follow an average two-year reproductive cycle in this species, but like female F01, may sometimes be mated to harassing males just after parturition (Figure 2E-F; Table S3). Of the 20 genetically sampled females, only 4 used one of our sampled nurseries. Therefore, the other females either did not reproduce during the study period or gave birth at different, unsampled nursery locations (Table S4). Based on our parentage analysis and genotype reconstruction, a total of 41 adult breeders (20 males and 21 females; Table 1) were found in our system. Although this may be an underestimate due to unsampled nursery locations in our study area, the number of breeders is relatively low. Most litters (78%) had multiple sires showing the prevalence of polyandrous female mating as it is commonly found in reef sharks [19,20]. This proportion may be underestimated as the monogamous females had small, potentially incompletely sampled litters. Male reproductive success was highly skewed with few males siring litters on multiple occasions within and across years (Figure 3), potentially due to dominance hierarchy among them or migratory strategies during the mating season [22].

### Genetic diversity

The mean offspring internal relatedness (mean IR = 0.03) was not significantly higher than expected under random mating. However, maximum offspring IR values (IRmax = 0.50) were higher than those found in offspring of its sister species *Negaprion* *brevirostris* in the Bahamas (IRmax = 0.05 [48]). IR values were lower to that of full-siblings (mean IR = 0.14) in *Squalus* *acanthias* litters [49], although the estimates were limited to 7 microsatellite markers. Even the critically endangered *Pristis* *pectinata* did not reveal inbreeding (mean IR = -0.02 [50]), perhaps due to the absence of migration barriers of a continuous coastal environment. The same level of inbreeding was found in *Carcharhinus* *melanopterus* , another shark species of the Society Islands [21]. *Negaprion* *acutidens* clearly developed some inbreeding avoidance strategy by conducting specific behaviour including dispersal and migrations across the archipelago. Polyandry is often expected to effectively increase the cumulative genetic variation in a single litter and therefore decrease inbreeding. At the population level, this effect is likely to be mitigated by an increased variance in male reproductive success [51]. Additionally, studies on *Negaprion* *brevirostris* [19,48] suggested that high multiple paternity in lemon sharks is more likely a result of convenience polyandry than of indirect genetic benefits such as inbreeding avoidance. Therefore, it remains unlikely that polyandry alone play a major role in inbreeding avoidance mechanisms.

Considering these behaviours and the mating system displayed by sicklefin lemon sharks to avoid inbreeding, the observed degree of inbreeding is unusual for mobile free-living marine species. Chapman et al. [50] argue that male-biased dispersal from their natal area as well as no evidence that certain males dominate paternity may reduce the likelihood of inbreeding in K-selected elasmobranchs. These life history characteristics and reproductive behaviour are less evident for *Negaprion* *acutidens* in French Polynesia as: (1) a few males appear to dominate paternity (Figure 3) (2), some males are resident to an island and do not roam like other species do [22], and (3) although evidence for dispersal was found, individuals within the entire Archipelago remain closely related (Figure 1). Another reason that may limit the effectiveness of inbreeding avoidance may be the fragmented habitat in this region which tends to isolate populations [26] and may reduce the opportunity to find unrelated mates.

**Figure 4:**
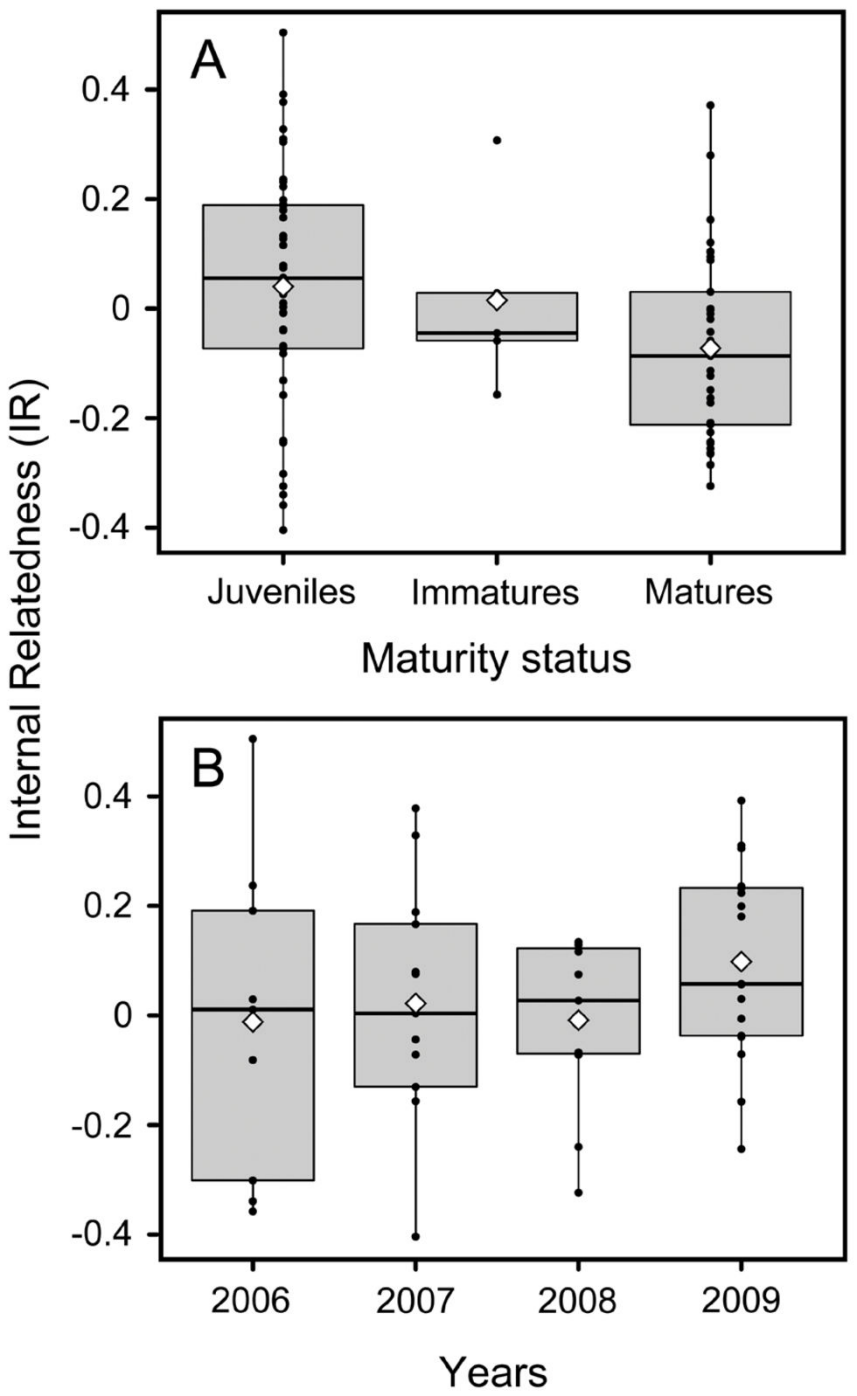
Patterns of distribution in internal relatedness values (IR). (A) IR values across the maturity stage of individuals (categories: juvenile < 100 cm, immature = 100-199 cm and mature > 200 cm). (B) IR values of newborn sharks (cohorts) across years. Box plots show the median (line within the boxes), mean (white diamond) and interquartile ranges IQR (boxes). Raw data points are indicated by black circles.

In addition, significantly higher IR values in juveniles than in adults (Figure 4; Figure S2) demonstrate a temporal change in inbreeding levels. Such a change, that suggests variation in fitness through time can result either from individuals with higher IR values progressively migrating away from their natal site or some differential mortality with individuals with higher IR values showing lower survival rate than juveniles with higher genetic diversity. Longer monitoring and redundancy of such pattern in inbreeding values will be necessary to determine which hypothesis should be favored.

## Conclusion

Individuals from this local population of sickefin lemon sharks in the Society archipelago share many first-order genetic relationships providing evidence that population size in this species is fairly limited in the context of islands at least in French Polynesia, but likely in isolated islands system of the Pacific. This low genetic variability has encouraged sicklefin lemon sharks to develop different strategies to avoid mating between close relatives with evidence of migrations across islands and atolls. The highly isolated and fragmented environment of French Polynesia [26] may limit encounter rate of unrelated mates despite dispersal and migrations across the Society Islands. These results encourage for long-term monitoring to survey the population response to increasing anthropogenic factors [22] despite the economic importance of these sharks in the local tourism industry [24].

## Supporting Information

Figure S1 **Sampled nursery locations.** (A) Map of French Polynesia with sampled islands. (B) Sampled nursery locations in Tetiaroa. (C) Nursery locations in Moorea. Circles are coloured according to the nursery location used in the study and black circles refers to adult sampling sites.
(TIF)

Figure S2 **Distribution of internal relatedness values (IR) of adult (black bars) and juvenile (grey bars) lemon sharks.**
(TIF)

Table S1 **Description of the 16 microsatellite loci used to genotype sicklefin lemon sharks.** Dyes: fluorescent Beckman Coulter dyes labels; Ta: annealing temperature (°C); N: number of individual scored; H_0_: observed heterozygosity; H_E_: expected heterozygosity; k: number of alleles; Fis: inbreeding coefficient; H–W: exact test for departure from Hardy-Weinberg.
(DOCX)

Table S2 **Distribution of average relatedness values across categories (sex and socio-residency groups).** Mean relatedness is displayed together with SD in parenthesis.
(DOCX)

Table S3 **Underwater observations of male courtship behaviour in sicklefin lemon sharks in Moorea.** For each year, the females that each male was observed to follow is indicated.
(DOCX)

Table S4 **Underwater visual estimations of reproduction timing in sicklefin lemon sharks in Moorea.** DBW: Dermal Bite Wound (dates of observations are indicated); P: Parturition indicated in grey (estimated to have occurred during the time the female was absent from the observation area; dates of disappearance for pregnant females are indicated when available).
(DOCX)

Video S1 **Courtship behavior with female F01 is closely followed by male M10 in 23 October 2008.** Female F01 was in near to term gestation in August 2008 (Figure 2E) and presumably gave birth between August and October 2008 (Table S4). This female returned to our study site on 8 October 2008, was seen followed by male M10 in a courtship and then joined by male M31 (Figure 2F). Note that both male M10 and female F01 are focused in their courtship behavior and do not pay attention to the diver.
(MP4)
