# Supplementary figures and images for: Genetic Network and Breeding Patterns of a Sicklefin Lemon Shark (Negaprion acutidens) Population in the Society Islands, French Polynesia
Source: PLoS One. 2013 Aug 13;8(8):e73899. doi: 10.1371/journal.pone.0073899 (PMC3742621; doi:10.1371/journal.pone.0073899)

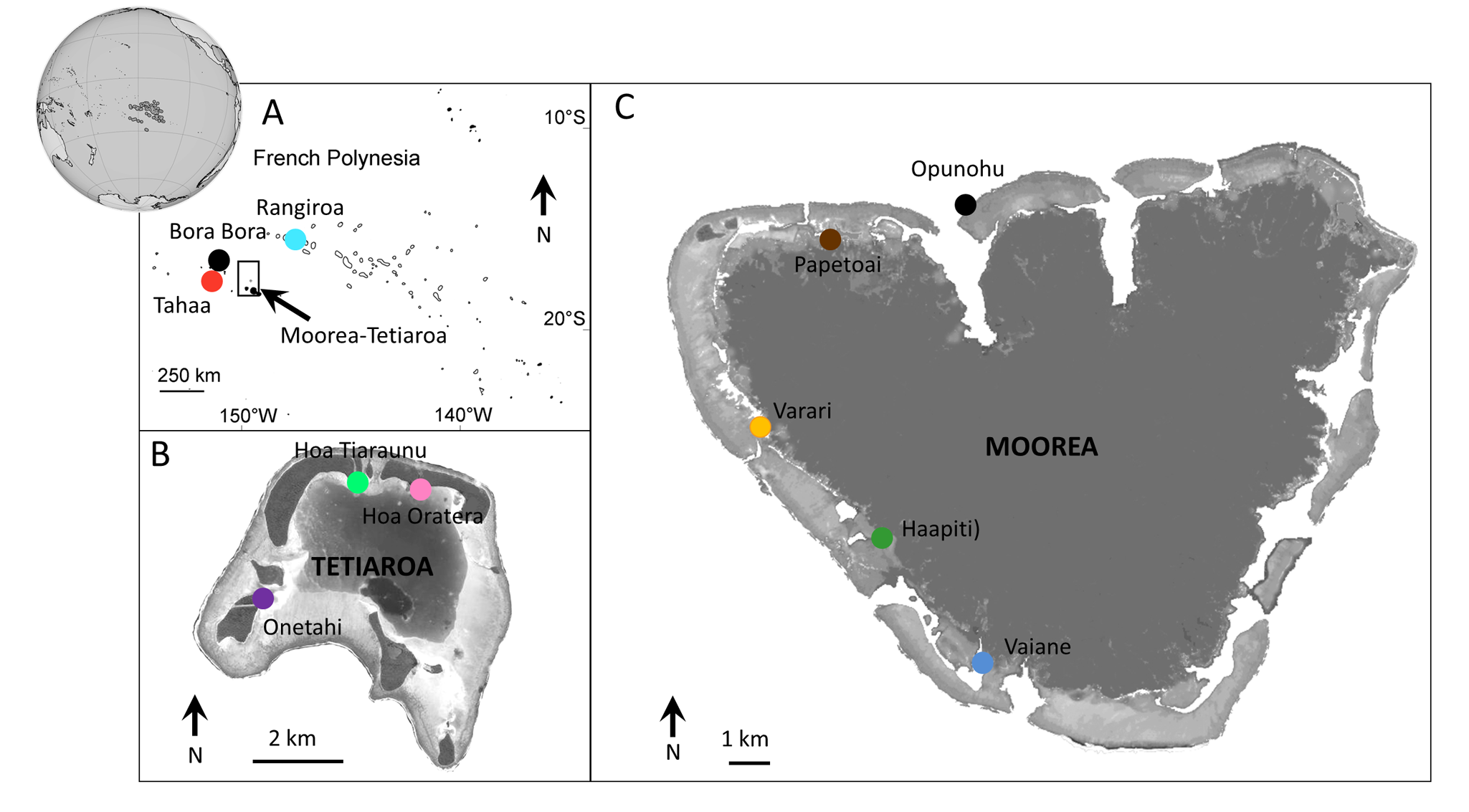

Supplement: Figure S1 — Sampled nursery locations. (A) Map of French Polynesia with sampled islands. (B) Sampled nursery locations in Tetiaroa. (C) Nursery locations in Moorea. Circles are coloured according to the nursery location used in the study and black circles refers to adult sampling sites. (TIF) [file pone.0073899.s001.tif]

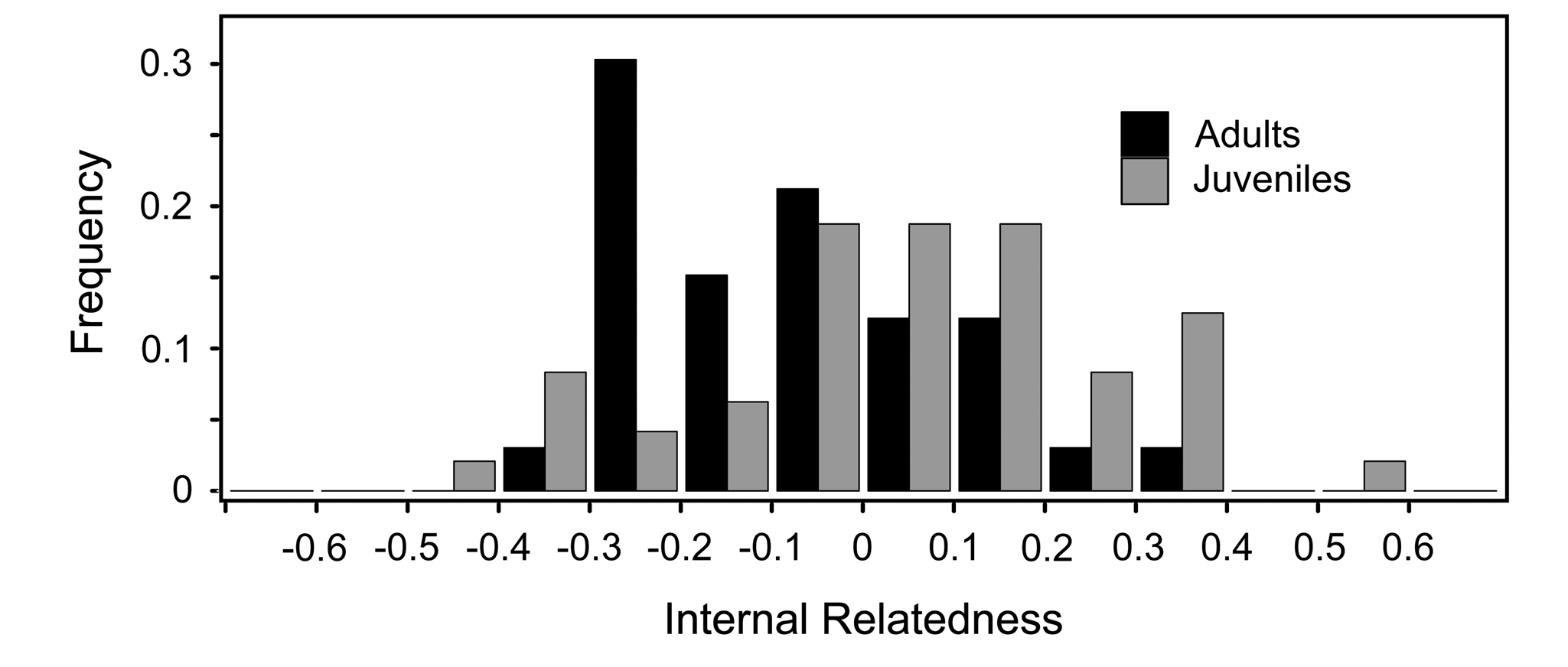

Supplement: Figure S2 — Distribution of internal relatedness values (IR) of adult (black bars) and juvenile (grey bars) lemon sharks. (TIF) [file pone.0073899.s002.tif]
