# Supplementary material for: Genetic Network and Breeding Patterns of a Sicklefin Lemon Shark (Negaprion acutidens) Population in the Society Islands, French Polynesia
Source: PLoS One. 2013 Aug 13;8(8):e73899. doi: 10.1371/journal.pone.0073899 (PMC3742621; doi:10.1371/journal.pone.0073899)
Supplement: Table S1 — Description of the 16 microsatellite loci used to genotype sicklefin lemon sharks. Dyes: fluorescent Beckman Coulter dyes labels; Ta: annealing temperature (°C); N: number of individual scored; H0: observed heterozygosity; HE: expected heterozygosity; k: number of alleles; Fis: inbreeding coefficient; H–W: exact test for departure from Hardy-Weinberg. (DOCX) [file pone.0073899.s003.docx]

**Table S1** **Description of the 16 microsatellite loci used to genotype sicklefin lemon sharks.** Dyes: fluorescent Beckman Coulter dyes labels; Ta: annealing temperature (°C); N: number of individual scored; H_0_: observed heterozygosity; H_E_: expected heterozygosity; k: number of alleles; Fis: inbreeding coefficient; H-W: exact test for departure from Hardy-Weinberg.

| **Locus** | **Primer sequence** **5'-3'** | **Repeat type** | **Dyes** | **Ta** | **N** | **H_0_** | **H_E_** | **k** | **F_is_** | **H-W** | **Source** |
| --- | --- | --- | --- | --- | --- | --- | --- | --- | --- | --- | --- |
| Na3 | F: GGCAGCCTTGCGTATTTACA | (CT)_5_(CA)_13_ | D3 | 57 | 85 | 0.412 | 0.395 | 3 | -0.0437 | NS | Current study |
|  | R: GGTAGTGGGAATCGACTGGA |  |  |  |  |  |  |  |  |  |  |
| Na6 | F: AGACGCATTGGTTGCCTAGT | (ATGG)_4_-(TAGA)_4_ | D2 | 57 | 85 | 0.435 | 0.456 | 2 | 0.0455 | NS | Current study |
|  | R: GAATCACCATCACCCACAAG |  |  |  |  |  |  |  |  |  |  |
| LS54 | F: TTGGAAACCGTGGAGGTGAA | (CT)_10_(CA)_8_ | D4 | 57 | 85 | 0.541 | 0.512 | 4 | -0.0576 | NS | [18] |
|  | R: GGGGAAAAAGAACTGGGACTAATCC |  |  |  |  |  |  |  |  |  |  |
| Cli102 | F: GACTGGCTGACCTAACTAAGC | (GA)_9_ | D3 | 57 | 85 | 0.624 | 0.687 | 4 | 0.0927 | NS | [52] |
|  | R: ATCCTGTGGTCCTTCTATC |  |  |  |  |  |  |  |  |  |  |
| LS11 | F: CCAGGAGAGAAGCATCTCACAG | (AC)_33_ | D2 | 60 | 85 | 0.776 | 0.635 | 3 | -0.2242 | NS | [53] |
|  | R: TGTCATTAGGATTTGCAGCC |  |  |  |  |  |  |  |  |  |  |
| LS53 | F: GCCTCATTCTGCTCCTGTGTTTT | (AC)_14_ | D4 | 64 | 85 | 0.388 | 0.406 | 3 | 0.0440 | NS | [53] |
|  | R: CACATAACCTCCTCCTCTGCTTCC |  |  |  |  |  |  |  |  |  |  |
| LS32 | F: TTAAGTCAGGCTATTGTGGACTCGT | (AC)_4_(AG)2(AC)_7_ | D3 | 64 | 85 | 0.506 | 0.533 | 6 | 0.0513 | NS | [53] |
|  | R: GCTTGCTTTCACACCTACCCATTT |  |  |  |  |  |  |  |  |  |  |
| LS15 | F: TGCGTGGGTTGTTGTTTTGG | (AC)_20_ | D2 | 57 | 85 | 0.365 | 0.487 | 3 | 0.2982 | * | [53] |
|  | R: GCACCTTGGATAGTTTGAGCAGG |  |  |  |  |  |  |  |  |  |  |
| LS24 | F: GGATGTGTTAGTGAGGTGGTGAGTG | (AC)_12_ | D3 | 60 | 85 | 0.435 | 0.440 | 2 | 0.0115 | NS | [53] |
|  | R: AGGGCAGAGACAGCAGGGAATATC |  |  |  |  |  |  |  |  |  |  |
| Cli107 | F: GGATTCACAACACAGGGAAC | (GT)_14_ | D2 | 57 | 85 | 0.694 | 0.850 | 12 | 0.1847 | * | [53] |
|  | R: CTCATTCTTAGTTGCTCTCG |  |  |  |  |  |  |  |  |  |  |
| LS75 | F^1^: TGTTACTGGGCACTATTATTC | (TC)_11_(AC)_11_AG(AC)_10_ | D4 | 57 | 85 | 0.694 | 0.721 | 5 | 0.0369 | NS | [53] |
|  | R: GAGGTTATCTTTTCTGTGTAGT |  |  |  |  |  |  |  |  |  |  |
| Cs08 | F: GGCCATCAGTTTGCTTA | (CA)_28_ | D4 | 57 | 85 | 0.871 | 0.871 | 13 | 0.0003 | NS | [54] |
|  | R: AATCCAGTTCCATCTTCAATA |  |  |  |  |  |  |  |  |  |  |
| Ct05 | F: TCTACTCTATTTTCTGCCCAATTAC | (GT)_19_ | D3 | 57 | 85 | 0.776 | 0.725 | 11 | -0.0712 | NS | [54] |
|  | R: TTTGGTAAGGCCAACTCCAG |  |  |  |  |  |  |  |  |  |  |
| Cpl90 | F: GTTGTTGCCTTGTCTTTCAATCG | (AC)_24_ | D3 | 57 | 85 | 0.812 | 0.767 | 5 | -0.0587 | NS | [55] |
|  | R: TGTGTCACTGTGTCTCTGTGTGCC |  |  |  |  |  |  |  |  |  |  |
| Cpl166 | F: TGGACATGACAATTACAGCACAGG | (GT)_17_ | D4 | 57 | 85 | 0.824 | 0.870 | 15 | 0.0534 | NS | [55] |
|  | R: CTGTTTACAACTTCCCTGGAGTGC |  |  |  |  |  |  |  |  |  |  |
| Cpl169 | F: TGACACAACCATTTATTCCCACG | (TG)_42_ | D2 | 57 | 85 | 0.788 | 0.830 | 12 | 0.0512 | * | [55] |
|  | R: GGTTTCCTTGAGTGAAAGAGAGAGC |  |  |  |  |  |  |  |  |  |  |

^1^ Forward flanking region changed after detection of null alleles with original primer pair of [18]

H-W: significance tests (*: P < 0.05 after Bonferonni correction, NS: not significant)

**Note on isolation of species-specific markers:**

To isolate species-specific microsatellite loci from *Negaprion acutidens*, we followed the protocol of Glenn and Schable [56] with modifications. Approximately 5 μg of high molecular weight DNA was digested using the blunt-end cutting enzyme *Rsa*I for three hours. Resulting fragments containing microsatellite motifs were hybridized to biotinylated oligonucleotides and captured using magnetic beads (Dynal, Oslo, Norway). A T-tailed vector was created by first digesting a pZErO-2 vector (Invitrogen, Carlsbad, California) with the enzyme *Eco*RV (Promega, Madison, Wisconsin) and then adding a T-tail by performing a 2-hour extension (72°C) with 100mM dTTP and polymerase (Bioline, San Clemente, California). The fragments were amplified, ligated into the T-tailed vector, and used to transform α-Select Gold Efficiency Competent Cells (Bioline, Randolph, MA). Recombinant clones were selected at random, amplified and sequenced on an ABI 3100 Genetic Analyzer (Applied Biosystems, Foster City, CA) by the EPSCoR Sequencing Facility at the Hawaii Institute of Marine Biology.

**References**

1. Keeney DB, Heist EJ (2003) Characterization of microsatellite loci isolated from the blacktip shark and their utility in requiem and hammerhead sharks. Mol Ecol Res 3: 501-504.
2. Feldheim KA, Gruber SH, Ashley MV (2001) Population genetic structure of the lemon shark (*Negaprion* *brevirostris*) in the western Atlantic: DNA microsatellite variation. Mol Ecol 10: 295–303.
3. Ovenden JR, Street R, Broderick D (2006) New microsatellite loci for Carcharhinid sharks (*Carcharhinus tilstoni* and *C. sorrah*) and their cross-amplification in other shark species. Mol Ecol Notes 6: 415–418.
4. Portnoy D, McDowell JR, Thompson K, Musick JA, Graves JE (2006) Isolation and characterization of five dinucleotide microsatellite loci in the sandbar shark, *Carcharhinus plumbeus*. Mol Ecol Notes 6: 431–433.
5. Glenn TC, Schable NA (2005)  Isolating microsatellite DNA loci. Methods Enzymol 395: 202-222.
