# Supplementary material for: Genetic Network and Breeding Patterns of a Sicklefin Lemon Shark (Negaprion acutidens) Population in the Society Islands, French Polynesia
Source: PLoS One. 2013 Aug 13;8(8):e73899. doi: 10.1371/journal.pone.0073899 (PMC3742621; doi:10.1371/journal.pone.0073899)
Supplement: Table S2 — Distribution of average relatedness values across categories (sex and socio-residency groups). Mean relatedness is displayed together with SD in parenthesis. (DOCX) [file pone.0073899.s004.docx]

**Table S2 Distribution of average relatedness values across categories (sex and socio-residency groups).** Mean relatedness is displayed together with SD in parenthesis.

|  |  | Mean R (SD) |
| --- | --- | --- |
| Global | Overall | 0.08 (0.03) |
|  |  |  |
| Group | Moorea Resident | 0.07 (0.03) |
|  | Moorea NonResident | 0.09 (0.02) |
|  | Bora Bora | 0.04 (0.02) |
|  | Within | 0.10 (0.06) |
|  | Between | 0.06 (0.03) |
|  |  |  |
| Sex | Males | 0.09 (0.03) |
|  | Females | 0.07 (0.02) |
|  | Within | 0.07 (0.03) |
|  | Between | 0.09 (0.04) |
