# Supplementary material for: Genetic Network and Breeding Patterns of a Sicklefin Lemon Shark (Negaprion acutidens) Population in the Society Islands, French Polynesia
Source: PLoS One. 2013 Aug 13;8(8):e73899. doi: 10.1371/journal.pone.0073899 (PMC3742621; doi:10.1371/journal.pone.0073899)
Supplement: Table S3 — Underwater observations of male courtship behaviour in sicklefin lemon sharks in Moorea. For each year, the females that each male was observed to follow is indicated. (DOCX) [file pone.0073899.s005.docx]

**Table S3 Underwater observations of male courtship behaviour in sicklefin lemon sharks in Moorea.** For each year, the females each sharks was observed to follow is indicated.

| Males | 2006 | 2007 | 2008 |
| --- | --- | --- | --- |
| M03 |  |  | F27 |
| M04 |  | F01, F24, F29 | F15, F20, F25 F27 |
| M05 |  |  |  |
| M07 |  | F24 |  |
| M09 |  |  |  |
| M10 |  | F06 | F01, F20 |
| M12 |  | F13 | F20, F27 |
| M18 | F30 | F24 | F20 |
| M19 | F13 | F01, F06 | F27 |
| M28 |  |  |  |
| M31 | F30 | F01, F29 | F11 |
| M34 |  |  |  |
| M35 |  |  |  |
| M36 |  |  | F15 |
| M37 |  | F29 | F20 |
| M38 |  |  | F11 |
| M39 |  |  |  |
