# Supplementary material for: Genetic Network and Breeding Patterns of a Sicklefin Lemon Shark (Negaprion acutidens) Population in the Society Islands, French Polynesia
Source: PLoS One. 2013 Aug 13;8(8):e73899. doi: 10.1371/journal.pone.0073899 (PMC3742621; doi:10.1371/journal.pone.0073899)
Supplement: Table S4 — Underwater visual estimations of reproduction timing in sicklefin lemon sharks in Moorea. DBW: Dermal Bite Wound (dates of observations are indicated); P: Parturition indicated in grey (estimated to have occurred during the time the female was absent from the observation area; dates of disappearance for pregnant females are indicated when available). (DOCX) [file pone.0073899.s006.docx]

**Table S4 Underwater visual estimations of reproduction timing in sicklefin lemon sharks in Moorea.** DBW: Dermal Bite Wound (dates of observations are indicated); P: Parturition indicated in grey (estimated to have occurred during the time the female was absent from the observation area; dates of disappearance for pregnant females are indicated when available).

| Females | 2005 | | 2006 | | 2007 | | 2008 | | 2009 | |
| --- | --- | --- | --- | --- | --- | --- | --- | --- | --- | --- |
|  | DBW | P | DBW | P | DBW | P | DBW | P | DBW | P |
| F1 |  | 29 Aug-14 Oct | Oct |  |  | 14 Jul-29 Aug |  | 25 Aug-08 Oct |  |  |
| F2 |  |  |  |  |  |  |  | 28 Aug-29 Sept |  |  |
| F6 | 27 Oct |  | 10 Sept | 03 Sept-26 Sept |  |  |  | 17 Sept-09 Oct |  |  |
| F11 |  | 25 Aug-06 Sept | Sept |  |  | 27 Aug-24 Sept | 09 Oct |  |  | 27 Aug-17 Sept |
| F13 | Sept-Oct |  |  | 21 Sept-18 Oct |  |  |  | 08 Aug-14 Nov |  |  |
| F15 |  | 05 Aug-23 Aug | Aug-Nov |  |  | 08 Aug-31 Aug | 27 Sept |  |  | 08 Sept-18 Sept |
| F16 |  | 20 Aug-01 Oct | 04 Nov |  |  |  |  |  |  |  |
| F17 |  |  |  |  | 11 Oct |  |  |  |  |  |
| F20 |  | 24 Aug- 04 Sept | 10 Oct |  |  | 27 Aug-15 Sept | Oct |  |  | 18 Sept-08 Oct |
| F21 |  | 23 Aug-11 Oct |  |  |  |  |  |  |  |  |
| F23 |  |  |  |  |  | 26 Sept-24 Oct | 13 Oct |  |  | 19 Sept-10 Oct |
| F24 |  |  |  |  |  |  |  |  |  |  |
| F25 |  |  | 26 Oct |  |  |  |  |  |  | ?-13 Oct |
| F26 |  |  | Oct |  |  |  |  |  |  |  |
| F27 |  |  | Oct |  |  | 15 Sept-16 Oct |  |  |  |  |
| F29 |  |  |  |  | 23 Oct |  |  | 09 Oct-28 Oct |  |  |
| F30 |  | 26 Jul-06 Sept | Oct |  |  | 20 Aug-25 Sept |  |  |  | ?-03 Oct |
